# The Value of Sugar and the Effect of Smoking on Muscular Work

**Published:** 1894-10

**Authors:** 


### The Value of Sugar and the Effect of Smoking on Muscular Work.

As the result of a series of experimental researches in the Physiological Institute, Turin, as to the value of sugar and the effect of smoking on muscular work, Vaughan Harley (*Journal of Physiology*, vol. xvi, Nos. 1 and 2, March, 1894) has come to the following conclusions:

1. The periods of indigestion as well as the kinds of food taken have a marked influence on voluntary muscular energy.

2. Irrespective of the influence of good, there is a periodical diurnal rise and fall in the power of performing muscular work.

3. More work can be done after than before midday.

4. The minimum amount of muscular power is in the morning about 8 A. M., the maximum about 4 in the afternoon.

5. Regular muscular exercise not only increases the size and power of the muscles, but has the effect of markedly delaying the approach of fatigue.

6. The amount of work performed on a diet of sugar alone is almost equal to that obtained on a full diet, fatigue, however, setting in sooner.

7. In fasting large quantities of sugar (500 grs.) can increase the power of doing muscular work during thirty voluntary contractions from 26 to 33 per cent. while the total gain in a day's work may be 61 to 76 per cent., the time before fatigue sets in being also lengthened.

8. The effect of sugar is so great that, when added to a small meal, it can increase the muscular power during thirty contractions from 9 to 12 per cent., while the total increase in work may be from 6 to 39 per cent., the approach of fatigue being at the same time retarded.

9. When added to a large mixed meal sugar can increase the muscular power of thirty contractions 2 to 7 per cent., the increase in total work being 8 to 16 per cent., and a marked increase in the resistance to fatigue is shown.

10. Two hundred and fifty grammes of sugar taken in addition to a full diet increases the day's work ; the work accomplished during thirty voluntary muscular contractions shows a gain of from 6 to 28 per cent., the total day's work giving an increase of power 9 to 36 per cent., and the time before fatigue sets in being lengthened.

11. Moderate smoking, although it may have a slight influence in diminishing the power of doing voluntary muscular work, neither stops the morning rise nor, when done early in the evening, hinders the evening fall.

12, Sugar taken early in the evening is capable of obliterating the diurnal fall of muscular power that occurs at this time, and increases the resistance to fatigue.

